# Knee Pain and Low Back Pain Additively Disturb Sleep in the General Population: A Cross-Sectional Analysis of the Nagahama Study

**DOI:** 10.1371/journal.pone.0140058

**Published:** 2015-10-07

**Authors:** Kimihiko Murase, Yasuharu Tabara, Hiromu Ito, Masahiko Kobayashi, Yoshimitsu Takahashi, Kazuya Setoh, Takahisa Kawaguchi, Shigeo Muro, Hiroshi Kadotani, Shinji Kosugi, Akihiro Sekine, Ryo Yamada, Takeo Nakayama, Michiaki Mishima, Shuichi Matsuda, Fumihiko Matsuda, Kazuo Chin

**Affiliations:** 1 Department of Respiratory Medicine, Graduate School of Medicine, Kyoto University, Kyoto, Japan; 2 Center for Genomic Medicine, Graduate School of Medicine, Kyoto University, Kyoto, Japan; 3 Department of Orthopedic Surgery, Graduate School of Medicine, Kyoto University, Kyoto, Japan; 4 Department of Health Informatics, Kyoto University School of Public Health, Kyoto University, Kyoto, Japan; 5 Department of Psychiatry, Shiga University of Medical Science, Shiga, Japan; 6 Department of Medical Ethics and Medical Genetics, Kyoto University School of Public Health, Kyoto, Japan; 7 Medical Research Support Center, Graduate School of Medicine, Kyoto University, Kyoto, Japan; 8 Department of Respiratory Care and Sleep Control Medicine, Graduate school of medicine, Kyoto University, Kyoto, Japan; University of New South Wales, AUSTRALIA

## Abstract

**Introduction:**

Association of knee and low back pain with sleep disturbance is poorly understood. We aimed to clarify the independent and combined effects of these orthopedic symptoms on sleep in a large-scale general population.

**Methods:**

Cross-sectional data about sleep and knee/low back pain were collected for 9,611 community residents (53±14 years old) by a structured questionnaire. Sleep duration less than 6 h/d was defined as short sleep. Sleep quality and the presence of knee and low back pain were evaluated by dichotomous questions. Subjects who complained about knee or low back pains were graded by tertiles of a numerical response scale (NRS) score and a Roland-Morris disability questionnaire (RDQ) score respectively. Multivariate regression analyses were performed to determine the correlates of short sleep duration and poor sleep quality.

**Results:**

Frequency of participants who complained of the orthopedic symptoms was as follows; knee pain, 29.0%; low back pain, 42.0% and both knee and low back pain 17.6%. Both knee and low back pain were significantly and independently associated with short sleep duration (knee pain: odds ratio (OR) = 1.19, p<0.01; low back pain: OR = 1.13, p = 0.01) and poor sleep quality (knee pain: OR = 1.22, p<0.01; low back pain; OR = 1.57, p<0.01). The group in the highest tertile of the NRS or RDQ score had the highest risk for short sleep duration and poor sleep quality except for the relationship between the highest tertile of the RDQ score and short sleep duration.(the highest tertile of the NRS: OR for short sleep duration = 1.31, p<0.01; OR for poor sleep quality = 1.47, p<0.01; the highest tertile of the RDQ: OR for short sleep duration = 1.11, p = 0.12; OR for poor sleep quality = 1.81, p<0.01) Further, coincident knee and low back pain raised the odds ratios for short sleep duration (either of knee or low back pain: OR = 1.10, p = 0.06; both knee and low back pain: OR = 1.40, p<0.01) and poor sleep quality (either of knee or low back pain: OR = 1.61, p<0.01; both knee and low back pain: OR = 2.17, p<0.01).

**Conclusion:**

Knee and low back pains were independently associated with short sleep duration and poor sleep quality. Further, they additively increased the correlation with these sleep problems in the general population.

## Introduction

Several studies have suggested that short sleep duration is one of the risks for obesity, hypertension, glucose intolerance and cardiovascular diseases (CVD) in general populations [1–4]. Further, poor sleep quality is also correlated with these conditions, and it has been suggested that there were adverse synergistic effects from poor sleep quality and short sleep duration on these conditions [5–9]. Although how these conditions are associated with sleep duration and quality remains uncertain, it has been reported that both short sleep duration and poor sleep quality contributed to hypertension and CVD by increasing sympathetic tonus and activity of inflammatory pathways [10,11]. Further, other studies found that sleep deprivation increased body weight and insulin resistance by changing metabolic hormonal levels and calorie intake [2,12,13]. These results emphasized the importance of clarifying factors that cause short sleep duration or poor sleep quality. However, few studies have been performed to determine these factors. Even though we previously reported that gastroesophageal reflux disease (GERD) symptoms and unfavorable dietary habits were independent determinants of short sleep duration in the general population [14], other previous studies suggested that sleep is affected by numerous lifestyle and health-related factors [15–17]. Therefore, there may still be residual but important determining factors for sleep disturbance that have not yet been identified.

Individuals with knee pain or low back pain frequently complain of sleep problems [18,19] and the relation between sleep and these musculoskeletal pains has been investigated. The one month prevalence of knee pain and low back pain in the general population was reported to be 20 to 25% and 20 to 35%, respectively [20–23]. Wilcox et al and Sasaki et al found that the onset and maintenance of sleep were the main types of sleep disturbance affected by knee pain [24,25]. Bahouq et al reported that individuals with low back pain commonly reported insomnia [26].

On the other hand, concomitant knee and low back pain is common and the intensities of knee pain and low back pain are positively correlated with each other [27,28]. However, the combined associations of these two musculoskeletal pains with sleep disturbance have never been investigated in a large scale population sample.

Here, we hypothesized that knee pain and low back pain were independently and additively correlated with sleep duration and quality and conducted a cross-sectional study by analyzing a dataset of the Nagahama Prospective Cohort for Comprehensive Human Bioscience (Nagahama Study), which is a large-scale population-based cohort study in Japan. We also hypothesized that frequencies of these orthopedic symptoms linearly increased with decreasing sleep duration. Further, because the previous studies suggested that a higher intensity of knee or low back pain was associated with higher frequency of sleep disturbance [29–31], we also analyzed the scores of the questionnaires that were correlated with the severity of these musculoskeletal pains and evaluated the association between these scores and sleep parameters.

## Methods

### Study participants

This study cohort was recruited from the general population living in Nagahama city, a largely rural city of 125,000 inhabitants in Shiga prefecture, located in the center of Japan. Recruitment was performed via mass communication such as newspaper flyers, brochures and Internet homepages of the local government and citizen organizations from 2008 to 2010. Information sessions about the present study for residents were also held by researchers and city employees as needed.

The inclusion criteria were as following: 1) age 30 to 74 years old, 2) able to participate in the health examinations independently, 3) no difficulty in communication and 4) voluntarily deciding to participate in the project.

A total of 9,809 residents voluntarily participated in the study. We collected their blood samples and instructed them to answer a questionnaire on their clinical background such as medical history and life habits. The majority of these data was not obtained from 5 participants who were then excluded from the baseline dataset. As a result, data from 9,804 individuals were finally included into the baseline dataset of the Nagahama study and the analysis of these baseline data took place in 2013 and 2014. Then, individuals with malignant tumors that were under treatment and/or clinical follow-up (n = 152) and who did not complete the questionnaire required for the present analysis (n = 41) were further excluded from the present analysis.

All study procedures were approved by the ethics committee of Kyoto University Graduate School of Medicine and by the Nagahama Municipal Review Board. Written informed consent was obtained from all participants.

### Assessment of sleep duration and quality

Sleep duration was investigated by the following question: “On average, how many hours do you sleep per day?” Regularity of sleep schedule was also investigated by the following “yes-no” question: “Are your waking time and bed time regular?” Quality of sleep was assessed by the following “yes-no” question: “Do you sleep poorly?” Participants who answered “yes” were defined as having poor sleep quality.

### Assessment of knee and low back pain

The presence of knee pain was determined by the following “yes-no” question: “Do your knees hurt?”. For the participants who answered “yes”, severity was assessed by a numerical response scale (NRS). In brief, participants who indicated the presence of knee pain were instructed to choose a number from 0 to 100 that represented pain intensity, with 0 indicating ‘no pain’ and 100 indicating ‘pain as bad as it could be’ [32]. Participants with knee pain were subdivided into three groups according to tertiles of the NRS score. Tertiles were designated as follows according to the degree of pain with higher scores indicating greater pain: T1-NRS,T2-NRS and T3-NRS. Those without knee pain were designated as the “no knee pain” group.

Low back pain was evaluated by the following “yes-no” question: “Does your low back hurt?”. Those participants who answered “yes” were instructed to respond to the Roland-Morris disability questionnaire (RDQ). RDQ is a well-validated and widely used scale that includes 24 items asking about low back pain related disability experienced during daily living. Each item in the RDQ can be answered with “yes” or “no”. The RDQ score is indicated by the sum of “yes” answers with a maximum of 24 points and higher scores represents more severe disability that is related to low back pain [33]. Participants with low back pain were categorized into 3 groups according to tertiles of the RDQ score: T1-RDQ, T2-RDQ and T3-RDQ. Participants without low back pain comprised the “no low back pain” group.

The RDQ includes the following question asking the direct relation between low back pain and sleep quality: “I sleep less well because of my back”. Because it was possible that the answer for this question considerably affected the analysis of the correlation between low back pain and sleep parameters, we calculated a modified RDQ score by excluding this item. We subdivided subjects again by tertiles of this modified RDQ score and performed the same analysis.

### Clinical parameters and life habits

Basic clinical parameters were also measured at baseline. Smoking and drinking habits and medical regimen were obtained by a structured questionnaire. Individuals who consumed alcohol more than 4 days/w were defined as frequent drinkers. With regard to the assessment of the medical regimens that were related to sleep and pain, the participants were questioned whether they were regularly taking “hypnotic drugs” or “analgesics”.

Details on methods of assessment of GERD symptoms and dietary behaviors were described in our previous report [14]. Briefly, GERD symptoms were evaluated using the “Frequency Scale for the Symptoms of GERD”, a well-validated and widely used questionnaire. Unfavorable dietary behaviors were assessed by the 4 “yes-no” questions about timing of meals and snacks and a score of one was assigned to each “yes” response.

### Statistical analysis

The participants were categorized into 5 groups according to sleep duration: less than 5 h, 5 to less than 6 h, 6 to less than 7 h, 7 to less than 8 h, and 8 or more h per day. Short sleep duration was defined as <6 h/d of sleep according to previous studies [3,14,34].

First, we divided the whole cohort into subgroups in 3 different ways according to gender, sleep duration and sleep quality, respectively. Differences in numeric variables among these subgroups were assessed by analysis of variance or Student’s t-test, while frequency differences were assessed by a chi-square analysis. In the comparison among subgroups categorized by sleep duration, trend analysis was also performed to assess whether target values showed increasing or decreasing trend as the sleep duration increased. These analyses were conducted by the Cochrane-Armitage trend test for categorical variables or the Jonckheere trend test for numerical variables. Further, to assess whether the presence of knee/low back pain was independently associated with short sleep duration or poor sleep quality, multiple logistic regression analysis of the whole cohort was performed. (Model 1) As we discuss later, since an irregular sleep schedule and taking hypnotics were strongly associated with sleep duration and quality, we conducted other multiple logistic regression analyses that included only participants without irregular sleep schedule (Model 2) or participants without taking hypnotics (Model 3) to confirm whether the significant association between sleep parameters and the musculoskeletal pains would remain.

Second, to examine the relationship between the severity of these orthopedic symptoms and sleep parameters, we performed multiple logistic regression analysis and calculated odds ratios, 95% confidence interval (CI) and p values for each subgroup categorized by tertiles of NRS scores for knee pain and RDQ scores for low back pain. The “no knee pain” or “no low back pain” subgroups served as references. Further, because this analysis showed an association between long sleep duration and a high RDQ score, we added the explorative analysis. We re-subdivided the whole cohort into 3 groups according to the sleep duration as follows: (1) short (less than 6h/d), (2) normal (6 to less than 8 h/d), (3) long (8 or more h/d). We then performed multinominal logistic regression analysis to confirm the correlation between sleep duration and RDQ score. Further, a multiple logistic regression analysis adopting modified RDQ score as described previously was also performed.

Lastly, to evaluate the additive association of knee and low back pain with sleep duration and quality, we categorized the present cohort into 3 groups according to the presence of knee and/or low back pain as follows: (1) neither knee nor low back pain, (2) either of knee or low back pain and (3) both knee and low back pain. Then, odds ratios in each group for short sleep duration and poor sleep quality were calculated by multiple logistic regression analysis with the group having neither knee nor low back pain as the reference.

In all multiple logistic regression analysis, the explanatory variables entered into the analysis were those yielding a p value <0.05 in the comparisons among subgroups categorized by sleep duration or sleep quality. Although collinearity between two independent explanatory variables was evaluated after converting the dichotomous variables to dummy variables, no strong collinearity (γ >0.7) was found in any combination of these explanatory variables.

All statistical analyses were performed using JMP (ver 11, SAS Institute Inc., Cary, NC, USA) or SPSS (ver 22, SPSS Japan, Inc., Tokyo, Japan) software, with a two-tailed p-value less than 0.05 considered to indicate statistical significance.

## Results

### Participants’ background

Characteristics of study participants are summarized in Table 1. Women complained of knee pain more frequently than men (women 30.6 vs. men 25.9%, p<0.01), while low back pain was less common in women (women 40.1 vs. men 46.0%, p<0.01). Participants with knee pain were significantly older (59±12 vs. 51±14 y, p<0.01), had a higher body mass index (BMI) (23.0±3.4 vs. 22.0±3.2 kg/m^2^, p<0.01), and were more frequently taking analgesic (5.3 vs. 2.5%, p<0.01) and hypnotic (8.1% vs. 4.2%, p<0.01) drugs than individuals without knee pain. The participants with low back pain were also older (55±13 vs. 53±13 y, p<0.01) and had higher BMI (22.7±3.4 vs. 22.0±3.2 kg/m^2^, p<0.01) than those without low back pain. The number of participants who were taking analgesic or hypnotic drugs was higher in those with than without low back pain. (4.8 vs. 2.2%, p<0.01 and 7.3 vs. 4.1%, p<0.01, respectively)

**Table 1:** Clinical characteristics of study participants.

	All	Men	Women	
	(n = 9,611)	(n = 3,157)	(n = 6,454)	p*
Age, y	53 ± 14	55 ± 14	52 ± 13	<0.01
Body mass index, kg/m^2^	22.3 ± 3.3	23.4 ± 3.1	21.7 ± 3.2	<0.01
Current smoker, %	14.7	31.2	6.6	<0.01
Frequent alcohol drinker, %	22.9	49.8	9.7	<0.01
Irregular sleep schedule, %	10.6	13.7	9.2	<0.01
Sleep medication, %	5.3	4.9	5.6	0.19
Analgesic drug, %	3.3	1.7	4.1	<0.01
GERD, %	22.9	22.5	23.1	0.52
No. unfavorable dietary behaviors	0.8 ± 0.9	1.0 ± 0.9	0.7 ± 0.8	<0.01
Knee pain, %	29.0	25.9	30.6	<0.01
Group categorized by the tertile of NRS score, %				
T1-NRS (NRS score ≥0 and <10)	12.4	10.8	13.1	
T2-NRS (NRS score ≥10 and <30)	9.6	8.5	10.2	
T3-NRS (NRS score ≥30)	7.1	6.6	7.3	
Low back pain, %	42.0	46.0	40.1	<0.01
Group categorized by the tertile of RDQ score, %				
T1-RDQ (RDQ score = 0)	12.1	13.7	11.4	
T2-RDQ (RDQ score = 1 or 2)	14.3	16.2	13.4	
T3-RDQ(RDQ score ≥3)	15.6	16.1	15.3	
Short sleep duration, %	29.4	25.9	31.1	<0.01
Poor sleep quality, %	17.6	17.2	17.8	0.46

Values are expressed as mean ± standard deviation or percentage.

GERD: gastroesophageal reflux disease, NRS: numerical response scale, RDQ: Roland-Morris Disability Questionnaire.

* p value in comparison with men and women.

Participants with knee pain were subdivided into 3 groups according to tertiles of the NRS score. Groups in the first, second and third tertile of NRS were designed as T1-NRS, T2-NRS and T3-NRS, respectively.

Participants with low back pain were subdivided into 3 groups according to tertiles of the RDQ score. Groups in the first, second and third tertile of RDQ were designed as T1-RDQ, T2-RDQ and T3-RDQ, respectively.

A substantial number of participants, specifically 60.6% of those with knee pain (corresponding to 17.6% of total participants), reported having both knee and low back pain.

### Comparisons among subgroups categorized by sleep parameters and the association between sleep parameters and knee/low back pain

Differences in characteristics by sleep duration are summarized in Table 2. Frequencies of females, irregular sleep schedule and consumption of hypnotic or analgesic drugs, BMI, GERD symptoms and the number of unfavorable dietary behaviors increased linearly with decreasing sleep duration. On the other hand, frequency of current smokers and alcohol drinkers decreased linearly with decreasing sleep duration. Trend analysis demonstrated that frequencies of knee and low back pain also linearly increased with decreasing sleep duration. In addition, these orthopedic symptoms were more frequently found in those with than without poor sleep quality (Table 3). The multiple logistic regression analysis indicated that both orthopedic symptoms were independently associated with sleep duration and quality after adjustment for possible covariates, while the odds ratio of low back pain for short sleep duration was somewhat lower than that for poor sleep quality. (Tables 4 and 5)

**Table 2:** Clinical characteristics of participants according to sleep duration.

	less than 5 h	5 to less than 6	6 to less than 7	7 to less than 8	8 or more h	p	p
	(n = 590)	(n = 2,234)	(n = 3,710)	(n = 2,325)	(n = 752)	ANOVA or Chi-square	trend
Male, %	30.2	28.6	31.1	37.2	43.1	<0.01	<0.01
Age, y	54±13	54±13	53±13	54±14	53±15	0.60	0.33
Body mass index, kg/m^2^	22.8±3.6	22.4±3.3	22.3±3.2	22.1±3.2	22.3±3.5	<0.01	<0.01
Current smoker, %	14.1	13.5	13.8	15.8	19	<0.01	<0.01
Frequent alcohol drinker, %	17.5	21.6	21.3	25.1	31.5	<0.01	<0.01
Irregular sleep schedule, %	25.9	14.7	8.4	6.8	9.4	<0.01	<0.01
Medication, %							
Hypnotic drugs	9.5	7.0	4.1	4.6	5.6	<0.01	<0.01
Analgesic drugs	4.2	3.9	3.4	2.7	2.4	0.07	<0.01
GERD, %	30.2	25.3	22.6	20.1	20.5	<0.01	<0.01
No. unfavorable dietary behaviors	1.0±1.0	0.9±0.9	0.8±0.9	0.8±0.8	0.8±0.9	<0.01	<0.01
Knee pain, %	35.6	33.3	28.3	25.8	25.4	<0.01	<0.01
Group categorized by the tertile of NRS score, %							
T1-NRS	15.6	12.9	12.6	11.3	10.2		
T2-NRS	9.0	11.9	9.3	8.7	8.1		
T3-NRS	11.0	8.5	6.3	5.9	7.1		
Low back pain, %	49.0	45.3	41.4	38.2	41.9	<0.01	<0.01
Group categorized by the tertile of RDQ score, %							
T1-RDQ	12.0	12.6	12.6	11.6	10.5		
T2-RDQ	16.4	15.7	13.5	13.6	14.5		
T3-RDQ	20.5	17.1	15.3	13.0	16.9		
Poor sleep quality, %	40.3	24.2	15.1	11.0	12.5	<0.01	<0.01

Values are expressed as mean ± standard deviation or percentage.

GERD: gastroesophageal reflux disease, NRS: numerical response scale, RDQ: Roland-Morris Disability Questionnaire.

**Table 3:** Clinical characteristics of participants with or without poor sleep quality.

	Participants without poor sleep quality	Participants with poor sleep quality	
	(n = 7,922)	(n = 1,689)	p
Male, %	33.0	32.1	0.46
Age, years	53±13	56±13	<0.01
Body mass index, kg/m^2^	22.3±3.3	22.4±3.3	0.15
Current smoker, %	14.8	13.7	0.26
Frequent alcohol drink, %	23.0	22.3	0.56
Irregular sleep schedule, %	9.3	17.0	<0.01
Medication, %			
Hypnotic drugs	2.0	21.1	<0.01
Analgesic drugs	2.9	5.2	<0.01
Gastroesophageal reflux disease, %	20.3	35.2	<0.01
No. of unfavorable dietary behaviors	0.8±0.9	0.9±0.9	<0.01
Knee pain, %	26.9	39.2	<0.01
Group categorized by the tertile of NRS score, %			
T1-NRS	12.0	14.0	
T2-NRS	8.9	13.1	
T3-NRS	6.0	12.1	
Low back pain, %	39.2	55.5	<0.01
Group categorized by the tertile of RDQ score, %			
T1-RDQ	12.3	11.6	
T2-RDQ	13.6	17.4	
T3-RDQ	13.3	26.5	

Values are expressed as mean ± standard deviation or percentage.

Differences in numeric variables between 2 groups were assessed by the Student’s t-test, while frequency differences were assessed by a chi-square analysis.

NRS: numerical response scale, RDQ: Roland-Morris Disability Questionnaire.

**Table 4:** Multiple logistic regression analysis to determine the factors identifying participants with short sleep duration.

	Model 1 (n = 9,611)			Model 2 (n = 8,589)			Model 3 (n = 9,098)		
	odds ratio	95% CI	P	odds ratio	95% CI	p	odds ratio	95% CI	p
Male	0.71	0.63–0.80	<0.01	0.69	0.61–0.79	<0.01	0.70	0.62–0.80	<0.01
Age (y)	1.00	0.99–1.00	0.15	1.00	0.99–1.01	0.39	1.00	0.99–1.01	0.12
Body mass index (kg/m^2^)	1.02	1.00–1.03	0.01	1.02	1.00–1.03	0.04	1.02	1.01–1.04	<0.01
Current smoker	0.91	0.79–1.05	0.20	0.89	0.76–1.04	0.15	0.91	0.79–1.05	0.21
Frequent alcohol drinker	0.95	0.84–1.08	0.44	1.00	0.88–1.14	0.99	0.94	0.83–1.07	0.33
Irregular sleep schedule	2.24	1.95–2.56	<0.01	-	-	-	2.28	1.98–2.63	<0.01
Hypnotic drugs	1.60	1.32–1.93	<0.01	1.70	1.39–2.08	<0.01	-	-	-
Analgesics	1.11	0.87–1.41	0.41	1.06	0.81–1.37	0.66	1.15	0.88–1.49	0.30
Gastroesophageal reflux disease	1.16	1.04–1.29	<0.01	1.16	1.03–1.30	0.01	1.18	1.06–1.32	<0.01
No. unfavorable dietary behaviors	1.19	1.12–1.25	<0.01	1.19	1.13–1.26	<0.01	1.20	1.13–1.26	<0.01
Knee pain	1.19	1.07–1.32	<0.01	1.18	1.05–1.32	<0.01	1.17	1.05–1.30	<0.01
Low back pain	1.13	1.02–1.24	0.01	1.12	1.02–1.24	0.02	1.11	1.00–1.22	0.04

Model 1: Analysis including all subjects.

Model 2: Analysis including only participants without irregular sleep schedule.

Model 3: Analysis including only participants who did not take hypnotics drugs.

**Table 5:** Multiple logistic regression analysis to determine the factors identifying participants with poor sleep quality.

	Model 1 (n = 9,611)			Model 2 (n = 8,589)			Model 3 (n = 9,098)		
	odds ratio	95% CI	P	odds ratio	95% CI	p	odds ratio	95% CI	p
Male	0.96	0.83–1.11	0.56	0.99	0.84–1.16	0.88	0.96	0.82–1.11	0.56
Age (y)	1.01	1.01–1.02	<0.01	1.01	1.01–1.02	<0.01	1.01	1.01–1.01	<0.01
Body mass index (kg/m^2^)	0.98	0.96–0.99	0.04	0.97	0.95–0.99	<0.01	0.98	0.96–0.99	0.03
Current smoker	0.88	0.74–1.05	0.18	0.81	0.66–0.99	0.04	0.93	0.78–1.11	0.42
Frequent alcohol drinker	0.93	0.80–1.08	0.34	0.95	0.80–1.12	0.54	0.98	0.83–1.14	0.78
Irregular sleep schedule	1.92	1.63–2.27	<0.01	-	-	-	1.98	1.67–2.34	<0.01
Hypnotic drugs	11.30	9.22–13.92	<0.01	12.00	9.68–15.01	<0.01	-	-	-
Analgesics	1.24	0.93–1.64	0.14	1.25	0.91–1.69	0.17	1.28	0.94–1.74	0.11
Gastroesophageal reflux disease	1.86	1.64–2.11	<0.01	1.84	1.61–2.12	<0.01	1.86	1.63–2.11	<0.01
No. unfavorable dietary behaviors	1.15	1.08–1.23	<0.01	1.19	1.11–1.28	<0.01	1.16	1.09–1.24	<0.01
Knee pain	1.22	1.08–1.39	<0.01	1.27	1.10–1.46	<0.01	1.22	1.07–1.40	<0.01
Low back pain	1.57	1.39–1.76	<0.01	1.60	1.40–1.82	<0.01	1.58	1.39–1.78	<0.01

Model 1: Analysis including all subjects.

Model 2: Analysis including only participants without irregular sleep schedule.

Model 3: Analysis including only participants who did not take hypnotics drugs.

In the comparison among subgroups categorized by sleep duration, the frequency of an irregular sleep schedule was approximately three times higher in the highest group than in the lowest group. (Table 2) Further, it was twice as high in those with than without poor sleep quality. (Table 3) We performed the multiple logistic regression analysis for short sleep duration and poor sleep quality again excluding the participants with an irregular sleep schedule. Then, we confirmed that the significant associations between sleep parameters and these musculoskeletal symptoms remained. (Tables 4 and 5) Further, the frequency of taking hypnotics was approximately 10 times higher in the group with than without poor sleep quality. (Table 3) We performed multiple logistic regression analysis in only the participants who did not take hypnotics and this analysis also demonstrated independent associations between sleep parameters and knee pain or low back pain. (Tables 4 and 5)

### Relation between the groups categorized by NRS/RDQ tertiles and sleep parameters

**Fig 1:**
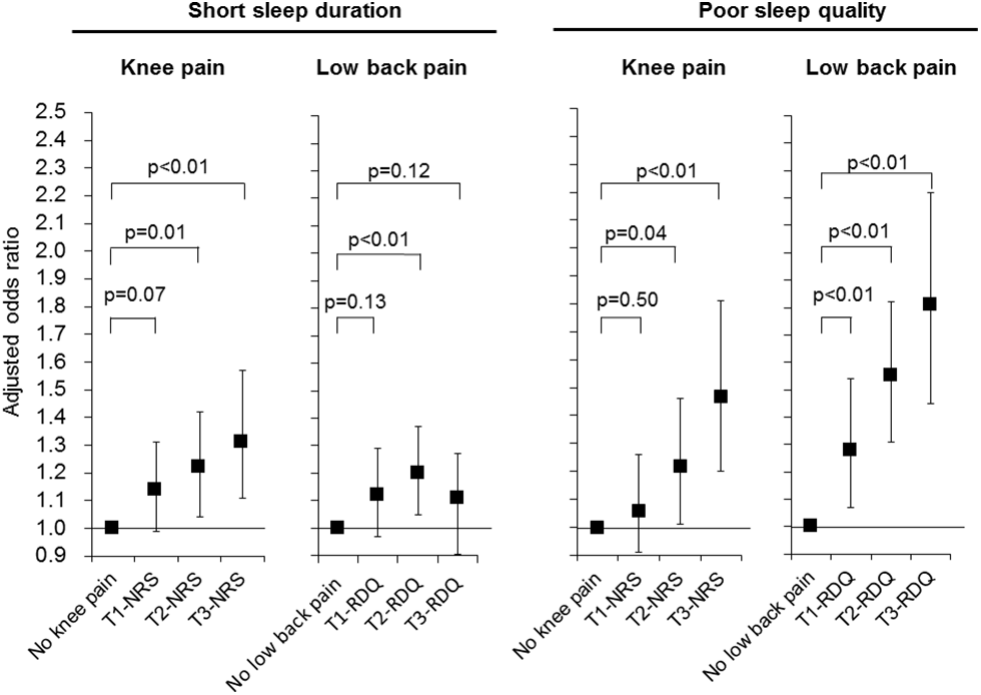
Odds ratios for short sleep duration and poor sleep quality in the subgroups categorized by tertiles of the NRS or RDQ score. Odds ratios and p values were calculated by multiple logistic regression analysis with the “no knee pain” or “no low back pain” subgroup as the reference. Adjusted factors in the regression model were as follows: age, body mass index, smoking status, frequent alcohol drinking, irregular sleep schedule, taking hypnotic or analgesic drugs, gastroesphageal reflux disease, the number of unfavorable dietary behaviors, and the categorization by the severity of other orthopedic symptom. Black squares and bars represent adjusted odds ratios and 95% confidence intervals, respectively. NRS: numerical response scale, RDQ: Roland-Morris Disability Questionnaire.

The T3-NRS group had highest odds ratio for short sleep duration and poor sleep quality. On the other hand, whereas the T3-RDQ group had the highest odds ratio for poor sleep quality, it was not associated with short sleep duration. (Fig 1) Although simple trend analysis showed that the overall frequency of low back pain was linearly increased with decreasing sleep duration (Table 2), the frequency of T3-RDQ was also high in the subgroup having a longer sleep duration (Table 2). Multinomial logistic regression analysis for short (<6 h/d), normal (6 h to <8h/d), and long (≥8 h/d) sleep duration demonstrated that T3-RDQ was slightly positively associated with long sleep duration (OR = 1.24, 95% confidence interval (CI) = 0.98–1.57, p = 0.08), while T2-RDQ was associated with short sleep duration (OR = 1.18, 95% CI = 1.03–1.36, p = 0.02)

We calculated a modified RDQ score and subdivided the participants by tertiles of the modified score. However, only a small number of participants (n = 13) were reclassified into different severity group. The multiple logistic regression analysis with this modified RDQ score did not show any substantial changes in the odds ratios of each group for short sleep duration and poor sleep quality, compared to the analysis using the original RDQ score.

### Combined association of knee/low back pain with sleep disturbance

**Fig 2:**
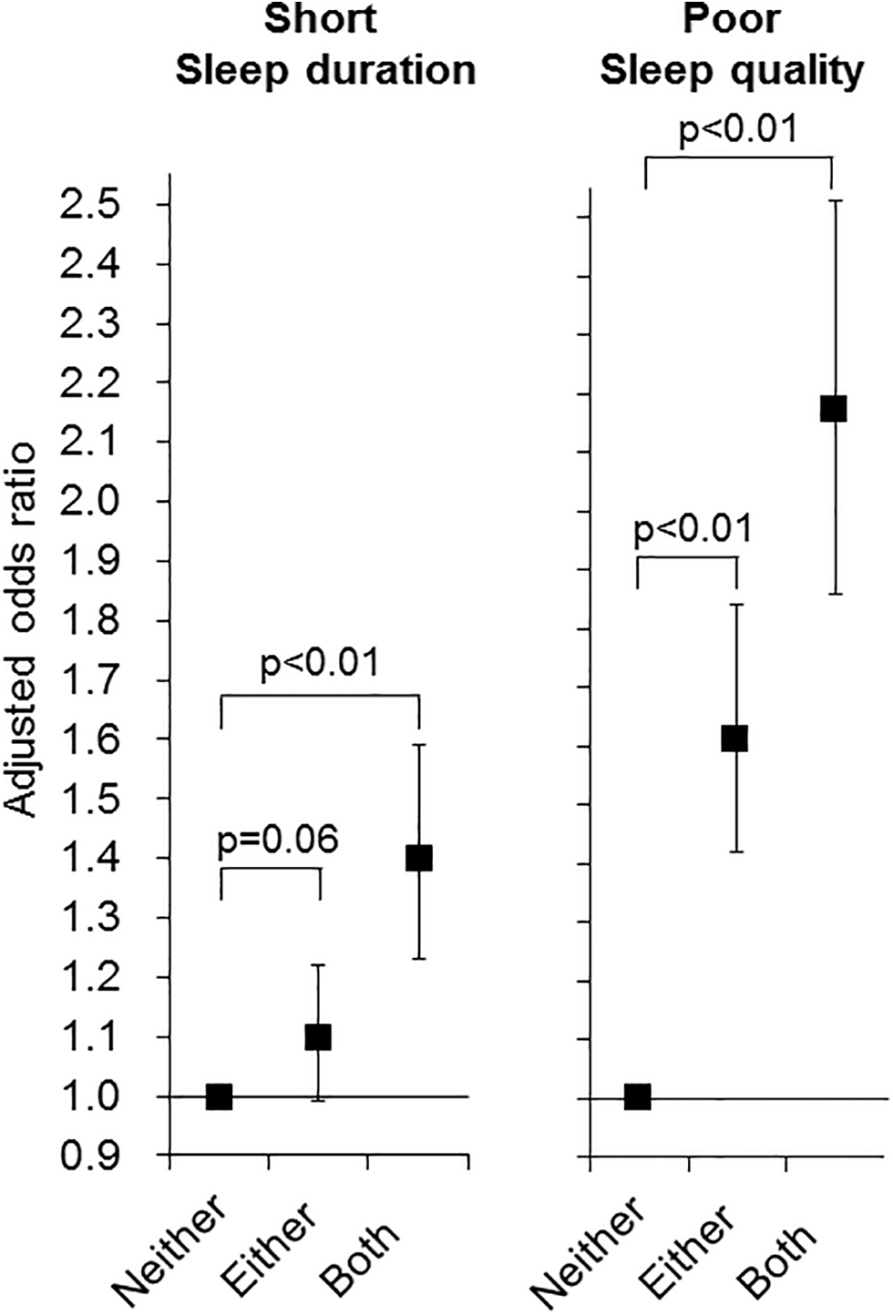
Odds ratios of coincident knee pain and low back pain for short sleep duration and poor sleep quality. Odds ratios and p values were calculated by multiple logistic regression analysis with the subgroup having neither knee nor low back pain as the reference. Adjusted factors in the regression model were as follows: age, body mass index, smoking status, frequent alcohol drinking, irregular sleep schedule, taking hypnotic or analgesic drugs, gastroesphageal reflux disease, and the number of unfavorable dietary behaviors. Black squares and bars represent adjusted odds ratios and 95% confidence intervals, respectively. Neither, neither knee nor low back pain; either, either knee or low back pain; both, both knee pain and low back pain.

Fig 2 depicts the results of multiple logistic regression analysis examining the combined association of knee and low back pain with short sleep duration and poor sleep quality. In either case, coincident knee and low back pain raised the odds ratios, though the relationship with poor sleep quality was stronger than that with short sleep duration.

## Discussion

The present study with a large sample from the general population demonstrated not only high concomitant frequency of knee and low back pain but also that knee and low back pain were significantly correlated with short sleep duration and poor sleep quality independently of other confounding factors. Further, concomitant knee pain and low back pain provided an additive risk for these types of sleep disturbance.

There was a high frequency of concomitance of knee and low back pain in the present study as in a previous study [27]. Because we did not specify the reasons for the knee or low back pain, we could not demonstrate the pathophysiologic mechanisms between these two orthopedic symptoms. Whereas the majority of patients with low back pain cannot be given a definitive diagnosis [35], some studies proposed the idea that low back pain was biomechanically linked to knee pain [36,37]. Further, another study suggested that these musculoskeletal pains reflected psychological conditions [38]. The mechanism of an association between these two musculoskeletal pains still remains elucidated.

A few studies have already investigated the relation between sleep disturbance and pain from several joints in large general population samples. Ohayon et al and Blay et al demonstrated that pain from several body parts was one of the correlates of sleep disturbance in 18,000 and 7,000 subjects, respectively [39,40]. However, they did not evaluate the combined effect of pain from a number of body parts on sleep. Concomitant knee and low back pain is common as already discussed, and the evaluation of their combined effects on sleep disturbance is clinically important. In fact, the present study demonstrated that both pains presented an additive risk for short sleep duration and poor sleep quality. These results suggested that taking into account not only the presence of knee or low back pain but also their concomitance is important in determining causes of an individual’s sleep complaints.

The present study also revealed that groups with higher tretile of NRS or RDQ score were associated with a greater risk of poor sleep quality. This indicates that evaluating not only the presence of joint pain but also the severity of pain and its related disability can help to determine the risk of sleep disturbance in each individual. On the other hand, the group with highest tertile of RDQ score was not associated with short sleep duration. Further, multinomial logistic regression analysis for (1) short (< 6h/d), (2) normal (6 h to <8h/d) and (3) long (8≥ h/d) sleep duration revealed a non-significant tendency for long sleep duration to predict membership of the T3-RDQ group (p = 0.08). The association between short sleep duration and T2-RDQ group membership remained statistically significant (p = 0.02). Even though the risk for short sleep duration was higher in those with than without low back pain in the analysis of the whole cohort (Table 4), the strength of the association between short sleep duration and low back pain may vary depending on the severity of the disability originating from low back pain or the severity of low back pain itself. In fact, while there has been a report of a high prevalence of sleep deprivation in patients with low back pain [41], other reports have suggested that long bed rest aggravated low back pain [42,43]. The relation between the severity of low back pain and sleep duration appears to be complicated and requires further investigations.

We evaluated sleep duration and sleep quality with a questionnaire. The analysis of the whole cohort showed a strong association between an irregular sleep schedule and short sleep duration. There might be a misperception of sleep duration in the group with an irregular sleep schedule as another study suggested [44]. However, in the analysis of the cohort without an irregular sleep schedule, knee and low back pain remained significant determinants of short sleep duration and poor sleep quality. On the other hand, the analysis also showed a strong association between taking hypnotics and poor sleep quality. Because a previous study reported that those with mental disorders or psychological distress were likely to be users of hypnotics [45], the participants taking hypnotics in the present cohort possibly had such clinical features. However, in the analysis that excluded participants who regularly took hypnotics, both knee and low back pain also remained significant correlates of sleep parameters. These findings enhanced the possibility that knee and low back pain were independent correlates of these sleep parameters from these confounding conditions.

Although the present study demonstrated that knee and low back pain were significant correlates of short sleep duration and poor sleep quality, the frequencies of specific sleep disorders such as sleep apnea and restless leg syndrome were not evaluated. Individuals with knee or low back pain may have a clinical background similar to those with sleep disorders. For example, obesity is the major correlate of both sleep apnea and musculoskeletal pains [46–49]. Lumbar radiculopathy is associated with knee and low back pain and also causes restless leg syndrome [50,51]. Therefore, knee and low back pain can be associated with these specific sleep disorders and clarifying these relationships would lead us to understand the more precise correlation between sleep disorders and musculoskeletal pains.

Our study has some limitations. First, because this study was based on a cross-sectional observation, we could not demonstrate a causal relationship between sleep disturbance and these musculoskeletal symptoms. Whereas pain prevents the initiation or the continuation of sleep [52,53], some studies suggested that sleep modulated the intensity of pain [54,55]. Therefore, a bi-directional relationship may be present between sleep disturbances and pain perception. The pathophysiological mechanisms between them should be clarified in future studies. Second, we used simple questions to assess sleep parameters. Sleep quality may be affected by multiple factors such as initiation and maintenance of sleep. Further, confounding factors which we could not evaluate may be present. For example, psychological distress and several social factors such as occupations and family composition of a family may affect sleep [56–59] but we did not evaluate these factors. Further studies using a well-validated questionnaire such as the Pittsburgh Sleep Quality Index [60] and questions examining psychological condition and social factors might better reveal the overall sleep-related problems and its determinants. Third, RDQ was established to assess the disability originating from low back pain, not the pain itself. Although a previous study showed that the RDQ score correlated well with the visual analogue scale score for low back pain [61], further studies investigating the relation between sleep and severity of low back pain itself are warranted. Lastly, we did not evaluate the character of the pain such as quality and duration. A recent study revealed a dose-response relation between the number of days with low back pain and risk of sleep disturbance [62]. Additional information on this issue will lead us to a more precise understanding of the correlation between sleep disturbance and pain.

## Conclusion

In summary, both knee pain and low back pain were associated with short sleep duration and poor sleep quality in the general population. Their concomitance and severity also contributed to sleep disturbance. The present study suggested the need for future studies clarifying the pathophysiological mechanisms between these musculoskeletal pains and sleep disturbance.
